# InCoB2014: mining biological data from genomics for transforming industry and health

**DOI:** 10.1186/1471-2164-15-S9-I1

**Published:** 2014-12-08

**Authors:** Christian Schönbach, Tin Wee Tan, Shoba Ranganathan

**Affiliations:** 1 Department of Biology, School of Science and Technology, Nazarbayev University, Astana 010000, Republic of Kazakhstan; 2 Center for AIDS Research, Kumamoto University, Kumamoto 860-0811, Japan; 3 Department of Biochemistry, Yong Loo Lin School of Medicine, National University of Singapore, Singapore 117599; 4 Department of Chemistry and Biomolecular Sciences and ARC Centre of Excellence in Bioinformatics, Macquarie University, Sydney NSW 2109, Australia

## Abstract

The 13th International Conference on Bioinformatics (InCoB2014) was held for the first time in Australia, at Sydney, July 31-2 August, 2014. InCoB is the annual scientific gathering of the Asia-Pacific Bioinformatics Network (APBioNet), hosted since 2002 in the Asia-Pacific region. Of 106 full papers submitted to the BMC track of InCoB2014, 50 (47.2%) were accepted in BMC Bioinformatics, BMC Genomics and BMC Systems Biology supplements, with three papers in a new BMC Medical Genomics supplement. While the majority of presenters and authors were from Asia and Australia, the increasing number of US and European conference attendees augurs well for the international flavour of InCoB. Next year's InCoB will be held jointly with the Genome Informatics Workshop (GIW), September 9-11, 2015 in Tokyo, Japan, with a view to integrate bioinformatics communities in the region.

## Introduction

The 13th InCoB (International Conference on Bioinformatics), an official conference of the Asia-Pacific Bioinformatics Network (APBioNet) [1], was held in Sydney, Australia. In keeping with InCoB's tradition to locate the conference in different Asia-Pacific countries, InCoB went to Australia, for the first time, with the NSW Chief Scientist and Engineer, Professor Mary O'Kane giving the opening address. The conference was well attended by the local community as well as participants from Asia-Pacific, Europe and USA, with scientists from Qatar and Turkey attending InCoB for the first time. The plenary talks covered mutations in DNA, proteomics, RNA Biology, systems biology, mathematics, statistics and computer science as well as bioinformatics training to equip scientists with the current "tools of the trade" and best practice methodologies, while the conference attendees presented cutting edge research with three industry presentations, 89 orals as well as 45 posters.

## Manuscript submission and review

We offered authors four tracks to submit manuscripts for potential publication in the supplement issues of BMC Bioinformatics, BMC Systems Biology or BMC Genomics (BMC track) and PeerJ [2]. Of the 112 submitted manuscripts, 106 were in the BMC track. All manuscripts received at least two reviews from the 92 member Program Committee, supported by 35 additional reviewers (Additional file 1). The first round of reviews resulted in the provisional acceptance of 12 (11.3%) manuscripts, with minor revisions. The authors of 45 manuscripts, including three that were transferred by the Program Committee Co-chairs to the PeerJ track, had to address major concerns raised by the reviewers. After a second round of review, another 38 (35.8%) manuscripts were provisionally accepted pending minor revisions. In all cases, the reviewers assessed the manuscripts at least twice and all submissions were ranked based on the reviewers' scores, in accord with earlier InCoB publications [3].

The 20 articles in this supplement cover mainly "genomic" topics, with three medically-oriented papers going into *BMC Medical Genomics *[4] for the first time. The InCoB2014 *BMC Bioinformatics *supplement [5] comprises 16 manuscripts while another 11 papers are presented in the *BMC Systems Biology *supplement [6]. Five more submissions have been published in the InCoB2014 PeerJ collection [7].

## Sequencing, genomes and genome analysis

With genome sequencing technologies becoming more and more accessible and affordable, the genetic origin of the Marwari horse was established by whole genome sequencing [8] while the chloroplast genome of Australia's macadamia nut tree [9]. funRNA [10] is a collection of RNA interference (RNAi) genes, implicated in genome defence as well as diverse cellular, developmental, and physiological processes, from fungal, metazoan and plant genomes along with bacterial and archaeal genomes. Abbas *et al.*[11] have assessed *de novo *assembly software for fungal genome data. Proteogenomics is increasingly used for the accurate annotation of protein coding regions using proteomic data. As currently available proteogenomic tools are tailored specifically for human and eukaryotic data, Uszkoreit *et al.*[12] have developed a proteogenomic analysis pipeline, specifically for bacterial genomes.

## Transcriptomics

Seven papers are devoted to transcriptome analysis addressing a range of challenges from epigenetics [13,14] to understanding transcript-level changes with diseases [15-18]. dCAP [13] is a new method to simultaneously detect constitutive and differential regulation of multiple epigenetic factors from multiple sample datasets, while the YNA database [14] provides an integrated data mining platform for chromatin changes in yeast. Huang *et al.*[15] have identified transcript-level changes implicated in biological dysfunction of energy metabolism and hemostasis in schizophrenia while Yarmishyn *et al.*[16] have pinpointed a non-coding RNA as a novel marker of neuroblastoma progression. Sheng *et al.*[17] have identified the most common microRNA (miRNA) editing events in colon cancer. For non-small cell lung cancer, Mah *et al.*[18] have identified a single single nucleotide polymorphism (SNP) as a good prognostic marker of patient outcome following chemotherapy. While transcripts are in the main used as a measure of protein expression, Sun *et al.*[19] have applied transcriptomics and pathway analysis to improve protein identification from proteomics data, especially where the sample size is limited to a few cells.

## Functional genomics

Guanine-rich nucleotide sequences form four-stranded G-quadruplex structures. Yano and Kato [20] have used hidden Markov Models to reliably identify these structures, especially those involved in DNA transcription. Wu *et al.*[21] have developed a novel algorithm (GM-SMCC), for predicting the functional annotation of protein-coding genes, applied successfully to the 2001 KDD cup yeast gene datasets. Small ubiquitin-like modifier (or SUMO) proteins covalently attached other proteins lead to sumoylation, which is involved in various cellular processes, including nuclear-cytosolic transport, transcriptional regulation, apoptosis, protein stability, response to stress, and cell cycle progression. Yavuz and Sezerman [22] have developed an accurate support vector machine (SVM)-based approach to identify these sites from sequence data, as a precursor to experimental validation.

## Pharmacogenomics

In the era of genomic medicine, it is possible that some approved drug molecules can be used for diseases other than those they were originally approved for. Yang and co-workers [23] propose the concept of "Homopharma", to combine similar drug binding environments to better understand molecular binding mechanisms for deploying approved drugs for other diseases, known as "repurposing." Tyagi *et al.*[24] have optimized arylthioindole compounds for efficiently disrupting tubulin assembly towards anti-cancer therapy, using QSAR and molecular dynamics approaches.

## Disease informatics

Taguchi and co-workers [25] have identified TINAGL1 and B3GALNT1 as novel key candidate genes in the treatment of non-small cell lung cancer from gene expression and epigenetic data, while sets of microRNA biomarkers for detecting lung squamous cell carcinoma have been proposed by Song *et al.*[26]. Xu *et al.*[27] have developed a novel methodology, MHC2MIL for developing peptide-based vaccines, benchmarked on 12 HLA DP and DQ molecules.

## Medical informatics

Papers specifically relating on genome-scale analysis with a disease focus are presented in a new supplement in BMC Medical Genomics and a brief overview of these articles is presented here. With pandemic viral infections spreading rapidly by jet travel, understanding cross-species transmissibility of vectors is addressed by Tan and co-workers [28] for influenza A, using a random forest approach. In the quest for diagnostic biomarkers and therapeutic targets, Nenadic and co-workers [29] have developed a novel text-mining approach to successfully identify novel genes and pathways for thyroid cancer subtypes. While gene-based cancer biomarkers are typically sets of hundreds or thousands of genes, Olsen *et al.*[30] have analysed public proteogenomic data to zoom in on just 32 tumor antigenic proteins as biomarkers for invasive ductal carcinomas. These studies point to the combined use of several "-omic" technologies as the focus of future studies for understanding, detecting and combatting diseases.

## Conclusion

With the growth in regional bioinformatics meetings, including ISCB-Asia meetings and the 2013 IEEE International Conference on Bioinformatics and Biomedicine (BIBM) in China [31], there is a growing community of bioinformatics scientists in the Asia-Pacific. To consolidate multiple meetings and to provide cross-talk between traditionally different bioinformatics communities, we invite you to attend the 2015 InCoB meeting to be held jointly with the Genome Informatics Workshop (GIW) in Tokyo, Japan [32].

## Competing interests

The authors declare that they have no competing interests.

## Authors' contributions

SR wrote the introduction. CS and SR (Program Committee Co-chairs) managed the review and editorial processes, respectively. TWT supported the post-acceptance manuscript processing.

## Supplementary Material

Additional file 1 **List of Program Committee Members and Additional Reviewers in Alphabetical Order**. Click here for file

## References

[B1] The Asia-Pacific Bioinformatics Network http://www.apbionet.org

[B2] PeerJ http://peerj.com

[B3] Schönbach C Shen B Tan T Ranganathan S InCoB2013 introduces Systems Biology as a major conference theme BMC Syst Biol 2013 7 Suppl 3 S1 10.1186/1752-0509-7-S3-S1 24555777 PMC3816296

[B4] Thirteenth International Conference on Bioinformatics (InCoB2014) Medical Genomics http://www.biomedcentral.com/bmcmedgenomics/supplements/7/S3

[B5] Thirteenth International Conference on Bioinformatics (InCoB2014) Bioinformatics http://www.biomedcentral.com/bmcbioinformatics/supplements/15/S16

[B6] Thirteenth International Conference on Bioinformatics (InCoB2014) Systems Biology http://www.biomedcentral.com/bmcsystbiol/supplements/8/S4

[B7] InCoB 2014 Collection - hosted on PeerJ https://peerj.com/collections/10-incob2014/

[B8] Jun J Cho YS Hu H Kim HM Jho S Gadhvi P Park KM Lim J Paek WK Han K Manica A Edwards JS Bhak J Whole genome sequence and analysis of the Marwari horse breed and its genetic origin BMC Genomics 2014 15 Suppl 9 S4 25521865 10.1186/1471-2164-15-S9-S4 PMC4290615

[B9] Nock CJ Baten AKM King GJ Complete chloroplast genome of *Macadamia integrifolia *confirms the position of the Gondwanan early-diverging eudicot family Proteaceae BMC Genomics 2014 15 Suppl 9 S13 25522147 10.1186/1471-2164-15-S9-S13 PMC4290595

[B10] Choi J Kim KT Jeon J Wu J Song H Asiegbu FO Lee YH funRNA: a fungi-centered genomics platform for genes encoding key components of RNAi BMC Genomics 2014 15 Suppl 9 S14 25522231 10.1186/1471-2164-15-S9-S14 PMC4290597

[B11] Abbas MM Malluhi QM Balakrishnan P Assessment of *de novo *assemblers for draft genomes: a case study with fungal genomes BMC Genomics 2014 15 Suppl 9 S10 25521762 10.1186/1471-2164-15-S9-S10 PMC4290589

[B12] Uszkoreit J Plohnke N Rexroth S Marcus K Eisenacher M The bacterial proteogenomic pipeline BMC Genomics 2014 15 Suppl 9 S19 25521444 10.1186/1471-2164-15-S9-S19 PMC4290607

[B13] Chen KB Hardison R Zhang Y dCaP: detecting differential binding events in multiple conditions and proteins BMC Genomics 2014 15 Suppl 9 S12 25522020 10.1186/1471-2164-15-S9-S12 PMC4290593

[B14] Hung PC Yang TH Liaw HJ Wu WS The Yeast Nucleosome Atlas (YNA) database: an integrative gene mining platform for studying chromatin structure and its regulation in yeast BMC Genomics 2014 15 Suppl 9 S5 25522035 10.1186/1471-2164-15-S9-S5 PMC4290617

[B15] Huang KC Yang KC Lin H Tsao TTH Lee SA Transcriptome alterations of mitochondrial and coagulation function in schizophrenia by cortical sequencing analysis BMC Genomics 2014 15 Suppl 9 S6 25522158 10.1186/1471-2164-15-S9-S6 PMC4290619

[B16] Yarmishyn AA Batagov AO Tan JZ Sundaram GM Sampath P Kuznetsov VA Kurochkin IV HOXD-AS1 is a novel lncRNA encoded in HOXD cluster and a marker of neuroblastoma progression revealed via integrative analysis of noncoding transcriptome BMC Genomics 2014 15 Suppl 9 S7 25522241 10.1186/1471-2164-15-S9-S7 PMC4290621

[B17] Zheng Y Li T Ren R Shi D Wang S Revealing editing and SNPs of microRNAs in colon tissues by analyzing high-throughput sequencing profiles of small RNAs BMC Genomics 2014 15 Suppl 9 S11 25521855 10.1186/1471-2164-15-S9-S11 PMC4290591

[B18] Mah TZ Yap XNA Limviphuvadh V Li N Srinath S Kuralmani V Feng M Liem N Adhikari S Yong WP Soo RA Maurer-Stroh S Eisenhaber F Tong JC Novel SNP improves differential survivability and mortality in non-small cell lung cancer patients BMC Genomics 2014 15 Suppl 9 S20 25521664 10.1186/1471-2164-15-S9-S20 PMC4290611

[B19] Sun J Zhang GL Li S Ivanov AR Fenyo D Lisacek F Murthy SK Karger BL Brusic V Pathway analysis and transcriptomics improve protein identification by shotgun proteomics from samples comprising small number of cells - a benchmarking study BMC Genomics 2014 15 Suppl 9 S1 25521637 10.1186/1471-2164-15-S9-S1 PMC4290587

[B20] Yano M Kato Y Using hidden Markov models to investigate G-quadruplex motifs in genomic sequences BMC Genomics 2014 15 Suppl 9 S15 25521044 10.1186/1471-2164-15-S9-S15 PMC4290599

[B21] Wu Q Ye Y Ho SS Zhou S Semi-supervised multi-label collective classification ensemble for functional genomics BMC Genomics 2014 15 Suppl 9 S17 25521242 10.1186/1471-2164-15-S9-S17 PMC4290603

[B22] Yavuz AS Sezerman OU Predicting sumoylation sites using support vector machines based on various sequence features, conformational flexibility and disorder BMC Genomics 2014 15 Suppl 9 S18 25521314 10.1186/1471-2164-15-S9-S18 PMC4290605

[B23] Chiu YY Tseng JH Liu KH Lin CT Hsu KC Yang JM Homopharma: a new concept for exploring the molecular binding mechanisms and drug repurposing BMC Genomics 2014 15 Suppl 9 S8 25521038 10.1186/1471-2164-15-S9-S8 PMC4290623

[B24] Tyagi C Gupta A Goyal S Dhanjal JK Grover A Fragment based group QSAR and molecular dynamics mechanistic studies on arylthioindole derivatives targeting the α-β interfacial site of human tubulin BMC Genomics 2014 15 Suppl 9 S3 25521775 10.1186/1471-2164-15-S9-S3 PMC4290613

[B25] Umeyama H Iwadate M Taguchi YH TINAGL1 and B3GALNT1 are potential therapy target genes to suppress metastasis in non-small cell lung cancer BMC Genomics 2014 15 Suppl 9 S2 25521548 10.1186/1471-2164-15-S9-S2 PMC4290609

[B26] Song R Liu Q Hutvagner G Nguyen H Ramamohanarao K Wong L Li J Rule discovery and distance separation to detect reliable miRNA biomarkers for the diagnosis of lung squamous cell carcinoma BMC Genomics 2014 15 Suppl 9 S16 25521201 10.1186/1471-2164-15-S9-S16 PMC4290601

[B27] Xu Y Luo C Qian M Huang X Zhu S MHC2MIL: a novel multiple instance learning based method for MHC-II peptide binding prediction by considering peptide flanking region and residue positions BMC Genomics 2014 15 Suppl 9 S9 25521198 10.1186/1471-2164-15-S9-S9 PMC4290625

[B28] Eng CLP Tong JC Tan TW Predicting host tropism of influenza A virus proteins using random forest BMC Medical Genomics 2014 7 Supp 3 S1 25521718 10.1186/1755-8794-7-S3-S1 PMC4290784

[B29] Wu C Schwartz JM Brabant G Nenadic G Molecular profiling of thyroid cancer subtypes using large-scale text mining BMC Medical Genomics 2014 7 Supp 3 S3 25521965 10.1186/1755-8794-7-S3-S3 PMC4290788

[B30] Olsen LR Campos B Winther O Sgroi DC Karger BL Brusic V Tumor antigens as proteogenomic biomarkers in invasive ductal carcinomas BMC Medical Genomics 2014 7 Supp 3 S2 25521819 10.1186/1755-8794-7-S3-S2 PMC4290786

[B31] BIBM 2013 http://bibm2013.tongji.edu.cn/

[B32] GIW-InCoB 2015 http://incob.apbionet.org/incob15

