# Supplementary material for: InCoB2014: mining biological data from genomics for transforming industry and health
Source: BMC Genomics. 2014 Dec 8;15(Suppl 9):I1. doi: 10.1186/1471-2164-15-S9-I1 (PMC4290585; doi:10.1186/1471-2164-15-S9-I1)
Supplement: Additional file 1 — List of Program Committee Members and Additional Reviewers in Alphabetical Order. [file 1471-2164-15-S9-I1-S1.pdf]

Additional File 1. List of InCoB2014 Reviewers (*.pdf)

We thank the members of InCoB2014 Scientific Program Committee and the external sub-reviewers (listed alphabetically below) for their valuable time, effort and constructive criticism of manuscripts submitted to InCoB2014 supplement issues of BMC Genomics, BMC Systems Biology or BMC Bioinformatics.

<u>InCoB2014 Scientific Program Committee:</u>

1. Shandar Ahmad (National Institute of Biomedical Innovation, Japan)
2. Tatsuya Akutsu (Kyoto University, Japan)
3. Shunsuke Aoki (Kyushu Institute of Technology, Japan)
4. Nicola Armstrong (Garvan Institute of Medical Research, Australia)
5. Vladimir Bajic (King Abdullah University of Science and Technology, Saudi Arabia)
6. Arsen Batagov (Bioinformatics Institute, A*STAR, Singapore)
7. Alex Bateman (European Bioinformatics Institute, UK)
8. Vladimir Brusic (Dana-Farber Cancer Institute, USA)
9. Zhi-Wei Cao (Shanghai Center for Bioinformatics Information Technology, China)
10. Filippo Castiglione (National Research Council of Italy, Italy)
11. Jonathan Chan (King Mongkut's University of Technology Thonburi, Thailand)
12. Ming Chen (Zhejiang University, China)
13. Jiajia Chen (Suzhou University of Science & Technology, China)
14. Wai-Ki Ching (The University of Hong Kong, SAR Hong Kong)
15. Ning Deng (Zhejiang University, China)
16. Frank Eisenhaber (Bioinformatics Institute, A*STAR, Singapore)
17. Mahmoud Elhefnawi (National Research Centre, Egypt)
18. Mohd Firdaus-Raih (Universiti Kebangsaan Malaysia)
19. Andrew French University of Nottingham, UK)

20. Ge Gao (Peking University, China)

21. Pascale Gaudet (Swiss Institute of Bioinformatics, Switzerland)

22. Charles Gilman (Nazarbayev University, Kazakhstan)

23. Kunde Ramamoorthy Govindarajan (Joint Genome Institute, USA)

24. Tim Hancock (Kyoto University, Japan)

25. Matthew He (Nova Southeastern University, USA)

26. Yongqun He (University of Michigan, USA)

27. Chia-Lang Hsu, National Taiwan University, Taiwan)

28. Guang Hu (Soochow University, China)

29. Chun-His Huang, University of Connecticut, USA)

30. Ming-Jing Hwang (Academia Sinica, Taiwan)

31. Chen Jiajia (Soochow University, China)

32. Ulykbek Kairov (Nazarbayev University, Kazakhstan)

33. Asif M. Khan (Perdana University, Malaysia)

34. Javed M. Khan (Western Australian Institute for Medical Research, Australia)

35. Tsung Fei Khang (University of Malaya, Malaysia)

36. Daisuke Kiga (Tokyo Institute of Technology, Japan)

37. Akira Kinjo (Osaka University, Japan)

38. Akihiko Konagaya (Tokyo Institute of Technology, Japan)

39. Shinji Kondo (National Institute of Polar Research, Japan)

40. Anton Kratz (RIKEN Omics Science Center, Japan)

41. Gaurav Kumar (Virgina Commonwealth University, USA)

42. Igor Kurochkin (Bioinformatics Institute, A*STAR, Singapore)

43. Chee Keong Kwoh (Nanyang Technological University, Singapore)

44. Jinyan Li (University of Technology, Sydney, Australia)

45. Guo-Zheng Li (Tongji University, China)

46. Xinghua Lu (University of Pittsburgh, USA)

47. Hiroshi Mamitsuka (Kyoto University, Japan)

48. Xizeng Mao (University of Georgia, USA)

49. Hideo Matsuda (Osaka University, Japan)

50. Bui Quang Minh (Max F. Perutz Laboratories, Austria)

51. Lenny Moise (University of Rhode Island, USA)

52. Santo Motta (University of Catania, Italy)

53. Kenta Nakai (The University of Tokyo, Japan)

54. Francesco Pappalardo (University of Catania, Italy)

55. Ashwini Patil (The University of Tokyo, Japan)

56. Nikolai Petrovsky (Flinders Medical Centre, Australia)

57. Jiang Qian (Johns Hopkins School of Medicine, USA)

58. Shoba Ranganathan (Macquarie University, Australia)

59. Yasubumi Sakakibara (Keio University, Japan)

60. Meena Sakharkar, Tsukuba University)

61. Daniele Santoni (National Research Council of Italy, Italy)

62. Arman Saparov (Nazarbayev University, Kazakhstan)

63. Christian Schönbach (Nazarbayev University, Kazakhstan)

64. Bairong Shen (Soochow University, China)

65. Tetsuo Shibuya (The University of Tokyo, Japan)

66. Narayanaswamy Srinivasan (Indian Institute of Science, India)

67. Durai Sundar (Indian Institute of Technology Delhi, India)

68. Y-H. Taguchi (Chuo University, Japan)

69. Yoichi Takenaka (Osaka University, Japan)

70. Martti Tammi (University of Malaya, Malaysia)
71. Tin Wee Tan (National University of Singapore, Singapore)
72. Weidong Tian (Fudan University, China)
73. Paolo Tieri (University of Bologna, Italy)
74. Joo Chuan Tong (IHPC, A*STAR, Singapore)
75. Sissades Tongsima (National Center for Genetic Engineering and Biotechnology, Thailand)
76. Ikuo Uchiyama (National Institute for Basic Biology, Japan)
77. Chandra Verma (Bioinformatics Institute, A*STAR, Singapore)
78. Mauno Vihinen (Lund University, Sweden)
79. Xiujie Wang (CAS, Institute for Genetics and Developmental Biology, China)
80. Yufeng Wang (University of Texas at San Antonio, USA)
81. Martin Wasser (Bioinformatics Institute, A*STAR, Singapore)
82. Dongqing Wei (Shanghai Jiaotong University, China)
83. Gonghong Wei (University of Oulu, Finnland)
84. Limsoon Wong (National University of Singapore, Singapore)
85. Jingfa Xiao (Beijing Institute of Genomics, China)
86. Chao Xie (National University of Singapore, Singapore)
87. Yu Xue (University of Science and Technology of China, China)
88. Yan Zhang (CAS Shanghai Institutes for Biological Sciences, China)
89. Guang Lan Zhang (Boston University, USA)
90. Xing-Ming Zhao Tongji University, China)
91. Xingming Zhao (Tongji University, China)
92. Shanfeng Zhu (Fudan University, USA)

External sub-reviewers:

1. Abdul Baten (Southern Cross University, Australia)
2. Peter Borger (University Hospital Basel, Switzerland)
3. Sarah Burge (European Bioinformatics Institute, UK)
4. Jobichen Chacko (National University of Singapore, Singapore)
5. Haifen Chen (Nanyang Technological University, Singapore)
6. Xing Chen (Academy of Mathematics and Systems Science, Chinese Academy of Sciences, China)
7. Austin W.T. Chiang (National Yang-Ming University, Taiwan)
8. Teresa Colombo (Istituto per le Applicazioni del Calcolo "Mauro Piccone", Italy)
9. Giovanni M. Farinella (Istituto per le Applicazioni del Calcolo "Mauro Piccone", Italy)
10. Soheil Feizi (Massachusetts Institute of Technology, USA)
11. Jing Hou (Université de Strasbourg, France)
12. Yongli Hu (National University of Singapore, Singapore)
13. Vasanthan Jayakumar (Keio University, Japan)
14. Cizhong Jiang (Tongji University, China)
15. Mayumi Kamada (Kyoto University, Japan)
16. Derin Keskin (Dana-Farber Cancer Institute, USA)
17. Hang-Mao Lee (University of Oulu, Finland)
18. Zhenhua Li (Peking University, China)
19. Yunchao Ling (Beijing Institute of Genomics, Chinese Academy of Sciences, China)
20. Mohd S. Mohamad (Universiti Teknologi Malaysia, Malaysia)
21. Maria C. Palumbo (Instituto per le Applicazioni del Calcolo "Mauro Piccone", Italy)
22. Vinca Prana (Instituto per le Applicazioni del Calcolo "Mauro Piccone", Italy)
23. C. Ramakrishnan (Indian Institute of Technology Madras, India)

24. Heiko Schmidt (University of Vienna, Austria) Alessandro Sette (La Jolla Institute for Allergy & Immunology, USA)
25. Philip Shaw (National Center for Genetic Engineering and Biotechnology, Thailand)
26. Cuong T. Quan (University of Tübingen, Germany)
27. Martin Vingron (Max Planck Institute of Molecular Genetics, Germany)
28. Quan Wang (Vanderbilt University, USA)
29. Tao Wang (Peking University, China)
30. Jiayan Wu (Beijing Institute of Genomics
31. Chinese Academy of Sciences, China)
32. Min Wu (Institute for Infocomm Research, A*STAR, Singapore)
33. Peng Yang (Institute for Infocomm Research, A*STAR, Singapore)
34. Yun Yu (Rice University, USA)
35. Yongbing Zhao (Beijing Institute of Genomics, Chinese Academy of Sciences, China).
